# Positive Psychology and Gratitude Interventions: A Randomized Clinical Trial

**DOI:** 10.3389/fpsyg.2019.00584

**Published:** 2019-03-21

**Authors:** Lúzie Fofonka Cunha, Lucia Campos Pellanda, Caroline Tozzi Reppold

**Affiliations:** ^1^Psychological Assessment Laboratory, Psychology Department, Federal University of Health Sciences of Porto Alegre (UFCSPA), Porto Alegre, Brazil; ^2^Community Health Department, Federal University of Health Sciences of Porto Alegre (UFCSPA), Porto Alegre, Brazil

**Keywords:** randomized clinical trial, positive psychology, gratitude, positive psychology interventions, well-being

## Abstract

**Objective:** The purpose of this study was to assess the effect of a gratitude intervention on a community sample of adults in relation to aspects involving well-being and mental health.

**Methods:** A randomized clinical trial was conducted with 1,337 participants, composed of an intervention group (Gratitude group, *n* = 446), and two control groups (Hassles group, *n* = 444 and Neutral Events group, *n* = 447). Participants assigned to the intervention condition were asked to write daily gratitude lists for 14 days, listing moments they had been grateful for during the day. The outcomes analyzed were affect, depression, happiness and life satisfaction. Participants completed the positive affect and negative affect schedule (PANAS), center for epidemiological studies depression scale (CES-D), subjective happiness scale (SHS), and satisfaction with life scale (SWLS) three times: pre- and post-intervention and at 14 days after the end of the intervention. Due to attrition, the number of participants analyzed was 410.

**Results:** Before the intervention, the groups did not differ in any of the variables examined, and loss to follow-up was random among the three groups. The gratitude intervention managed to increase positive affect, subjective happiness and life satisfaction, and reduce negative affect and depression symptoms. This change was greater than the changes in the control groups in relation to positive affect. In the other outcomes analyzed, similar changes were observed in the gratitude intervention and the neutral events intervention.

**Conclusion:** Some similarities were found between the Gratitude and the Neutral Events groups probably because participants in the last group usually recorded positive events from their days on the lists, turning it into an activity very similar to that proposed to the gratitude group. Some limitations of the study are discussed, such as the high dropout rate for self-performed online interventions. It is necessary to investigate which characteristics of an intervention ensure better results when the intervention is performed online.

**Trial Registration:** The study is registered in the Brazilian Clinical Trials Registry, under No. RBR-9j9myd. Trial URL: http://www.ensaiosclinicos.gov.br/rg/RBR-9j9myd/.

## Introduction

Scientific evidence suggests there is a relationship between gratitude and well-being (Wood et al., 2010). Subjective well-being is composed of two dimensions: one that is cognitive and the other emotional. The first is related to the individual’s level of satisfaction with life, and the second results from the prevalence of positive over negative affect (NA) (Diener, 1984).

In general terms, gratitude stems from the recognition that something good happened to you, accompanied by an appraisal that someone, whether another individual or an impersonal source, such as nature or a divine entity, was responsible for it (Emmons and Shelton, 2002; Watkins et al., 2009). The mechanism by which gratitude is related to well-being is uncertain (Emmons and Mishra, 2011), but since Emmons and McCullough (2003) started investigating the influence of gratitude interventions on well-being, the results were so inspiring that it sparked various other studies (Davis et al., 2016).

One of the most commonly researched interventions is the gratitude list (Emmons and McCullough, 2003), where individuals list three to five things they felt grateful for during the day. This is a simple and quick activity that can promote increased positive affect (PA) (Martínez-Martí et al., 2010), happiness (Mongrain and Anselmo-Matthews, 2012) and life satisfaction (Manthey et al., 2016) and decreased NA (Chan, 2013), stress (Kerr et al., 2015) and depression symptoms (Southwell, 2012). Previous research has also suggested that the practice of gratitude is related to prosociality (McCullough et al., 2002), relationship formation and maintenance (Algoe et al., 2008), physical health symptoms (Emmons and McCullough, 2003), and sleep (Wood et al., 2009).

The signs that gratitude interventions can contribute to well-being are promising, but these results must be interpreted with caution, due to methodological issues, primarily in regard to the type of the comparison groups used in the studies (Wood et al., 2010; Davis et al., 2016; Dickens, 2017). In addition, most of the studies are conducted in countries from North America and Europe. The study of other populations, with different cultures, socio-political contexts and even diverse geography and climate would add to the generability of the gratitude effects, and could provide important insights on these populations.

In view of this scarcity of gratitude intervention studies and in order to contribute to international research on positive psychology-based interventions in different cultural contexts, the objective of the present study was to assess, through a randomized clinical trial, the effect of a gratitude intervention on a sample of adults in relation to aspects involving well-being and mental health. The hypothesis was that we would observe greater increases in positive affect, subjective happiness and life satisfaction and a decrease in NA and depression symptoms when compared to the control groups. And that this change would be observed over time and would be greater than the changes observed in the control groups.

## Materials and Methods

A blind randomized clinical trial was performed for assessing outcomes, with a gratitude intervention group and two control groups: hassles and neutral events.

### Sample

A sample size calculation analysis was performed to determine the necessary sample size for implementing the research design with procedures similar to those presented by Emmons and McCullough (2003) and Martínez-Martí et al. (2010), i.e., analyses of variance with repeated measures with between and within design in relation to participants.

Based on both studies, a low effect size was estimated (η^2^ = 0.061). The significance level was set at 0.05 and power at 0.95. Non-probabilistic sampling by convenience was used and the study was closed when the established sample size of at least 315 participants (105 in each group) was achieved.

**Figure 1:**
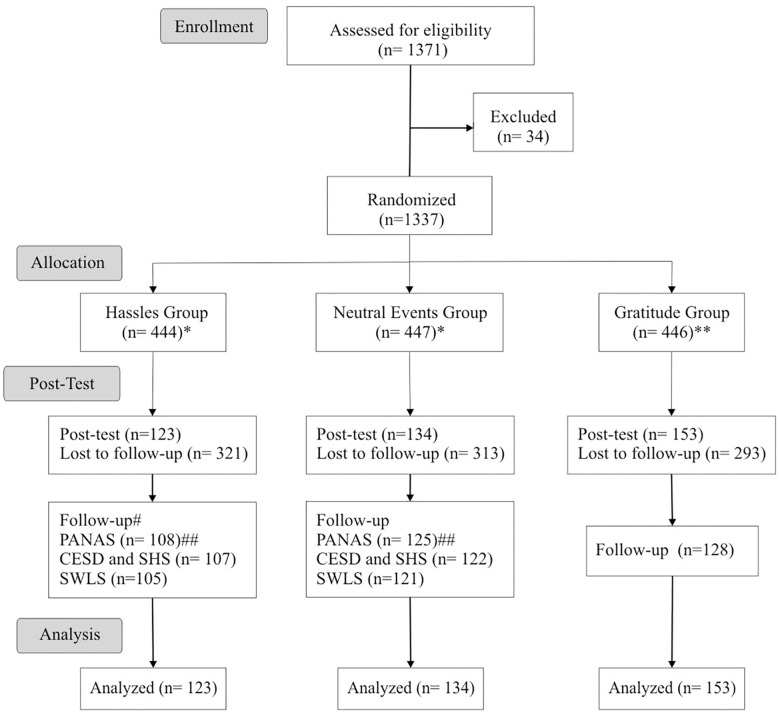
Inclusion, allocation, follow-up, and analysis flowchart. ^∗^Control group. ^∗∗^Intervention group. ^#^Measured two weeks after the end of the intervention. ^##^Number of participants who answered the respective scale.

The eligibility criteria were: participants over 18 years of age who voluntarily accepted to participate in the study and had an email for direct contact and daily Internet access. Thirty-four participants were excluded for providing incorrect or inaccessible emails, as shown in Figure 1.

### Instruments

The participants responded to the scales at three times: before the intervention (time 1), right after the intervention (time 2), and 14 days after the end of the intervention (time 3). The scales were filled in using the online SurveyMonkey questionnaire tool. Before the intervention, the participants also answered a questionnaire, with questions that enabled characterization of the sample. The questions included: the participant’s sex, region of the country where the person was born, marital status, number of children, average monthly family income, race/color of identification, and religion.

#### Positive and Negative Affect Schedule (PANAS) (Zanon and Hutz, 2014)

The positive and negative affect schedule is a scale composed of 20 items, where 10 assess PA and 10 assess NA. Each item refers to an adjective and the responses are classified in a 5-point Likert scale, according to how the person has been feeling the emotion described by the adjective lately. In a study conducted with 853 university students, the internal consistency of the scale was α = 0.83 for positive affects and α = 0.77 for negative affects (Zanon et al., 2013).

#### Center for Epidemiological Studies Depression Scale (CES-D) (Hauck Filho and Teixeira, 2011)

The center for epidemiological studies depression scale is a 20-item scale that assesses depression symptoms using a 4-point Likert scale. In a study by Hauck Filho and Teixeira (2011) conducted with 226 Brazilian university students, the internal consistency of the scale was α = 0.89.

#### Subjective Happiness Scale (SHS) (Damásio et al., 2014)

The subjective happiness scale is a scale that measures happiness and is comprised of 4 items, where the response options are structured in a Likert scale from 1 to 7. In a study conducted in Brazil with 600 individuals, the scale had a Cronbach’s alpha of 0.84 (Damásio et al., 2014).

#### Satisfaction With Life Scale (SWLS) (Hutz et al., 2014)

The satisfaction with life scale is a 5-item scale that assesses life satisfaction. The responses are classified in a 7-point Likert scale. In a study conducted with 1,388 Brazilian students, Cronbach’s alpha was 0.87 (Zanon et al., 2014).

### Procedures

It was divulged primarily among academic audiences of universities around the country, through email contact. All the data, including the scale responses and lists prepared daily, were collected through the SurveyMonkey online questionnaire tool. The informed consent form was drafted in a question and answer format, for easier understanding by the study participants.

The randomization was done through the Research Randomizer website in blocks of 3. According to the order of registration of participants in the study, they were allocated to one of three groups according to the randomization list generated by the website. Since it was a single-blind study, the participants were not aware of the existence of three groups in the study, or the group to which they had been randomized.

After the randomization stage, the participants received the instructions for the intervention. The participants were requested to set aside 10–20 min at the end of the day, before going to bed, for 14 days, to write the lists, according to their allocation group. In each group, the participants made a list of five items, assessing the activities from that day. The instructions for the groups were adapted and translated from studies by Emmons and McCullough (2003) and Martínez-Martí et al. (2010).

For the Hassles group, the instructions were:

In life, we sometimes encounter hassles and annoying situations that may bother and irritate us. They can occur in various realms of our lives (in personal relationships, in the workplace, at university, at home, or in relation to finances or health). Think back over the past day and write down five hassles or annoying situations that you had to face.

For the Neutral Events group, the instructions were:

During the day, there are events, both large and small, that end up affecting us. Think back over the past day and write down five events that somehow affected you.

For the Gratitude group, the instructions were:

There are many things in our lives, both large and small, that we might be grateful for. Think back over the past day and write down five things in your life that you are grateful for.

Every day, for 2 weeks, emails with a link were sent to the participants for them to respond to the online questionnaire. At the end of the 14 days, the participants were instructed to fill out the scales again for affect (PANAS), depression (CES-D), happiness (SHS), and life satisfaction (SWLS). Two weeks after the end of the intervention, they received another email to fill in the four scales once again.

### Statistical Analysis

The participants’ ages and the scale scores at time 1 were described using means and standard deviation, and the categorical variables by absolute values and frequencies. The comparison between the groups at time 1, and the participants remaining at time 2, was done through one factor analysis of variance (ANOVA) for the continuous variables (age and scale scores) and through Pearson’s chi-square test for the categorical variables (sociodemographic characteristics). The significance level used was 5%.

The Shapiro-Wilk Normality Test revealed that the variables had normal distribution. Loss to follow-up was assessed through Pearson’s chi-square test for the categorical variables and the independent *t*-test for the continuous variables.

Participants were analyzed according to their randomized group. Only participants who responded to the scales at time 2 or time 3 were included. Missing data was estimated using the expectation-maximization method. The outcomes analysis (affect, depression, happiness, and life satisfaction) was performed through an analysis by generalized estimating equations (GEE), where multiple adjusted comparisons by Bonferroni were done.

The significance level established for the study was α = 0.05. The interpretation of effect sizes used the proposal by Cohen (1988), where effect size was considered “small” when *d* = 0.2, “medium” when *d* = 0.5 and “large” when *d* = 0.8.

### Ethics Statement

This study was approved by the Research Ethics Committee of the Federal University of Health Sciences of Porto Alegre, RS, Brazil (Opinion No. 1.783.954), and is registered in the Brazilian Clinical Trials Registry, under No. RBR-9j9myd.

## Results

The flowchart (Figure 1) illustrates the random distribution of the participants in the different groups and during the study stages. In the period from November 2016 to September 2017, 1,371 individuals demonstrated interest in the study and 1,337 met the inclusion criteria and were randomized into three groups (Hassles = 444, Neutral Events = 447, Gratitude = 446). After randomization, 10 individuals wanted to withdraw from the study and 927 stopped sending the daily lists. The number of losses of participants during follow-up was the same among the three intervention groups (*p* = 0.095), i.e., the losses were random.

A total of 410 participants were analyzed (Hassles = 123, Neutral Events = 134, Gratitude = 153), and everyone completed all the scales at times 1 and 2. Participants who did not complete a scale at time 3 were kept in the analysis.

The participants who dropped out of the study were significantly younger than the participants who remained (*p* < 0.001). In relation to the scale scores, those who dropped out of the study had higher mean scores in the scales for NA (*p* = 0.008) and depression (*p* = 0.001) and lower mean scores in the scales for PA (*p* = 0.046), subjective happiness (*p* = 0.007) and life satisfaction (*p* < 0.001).

**Figure 2:**
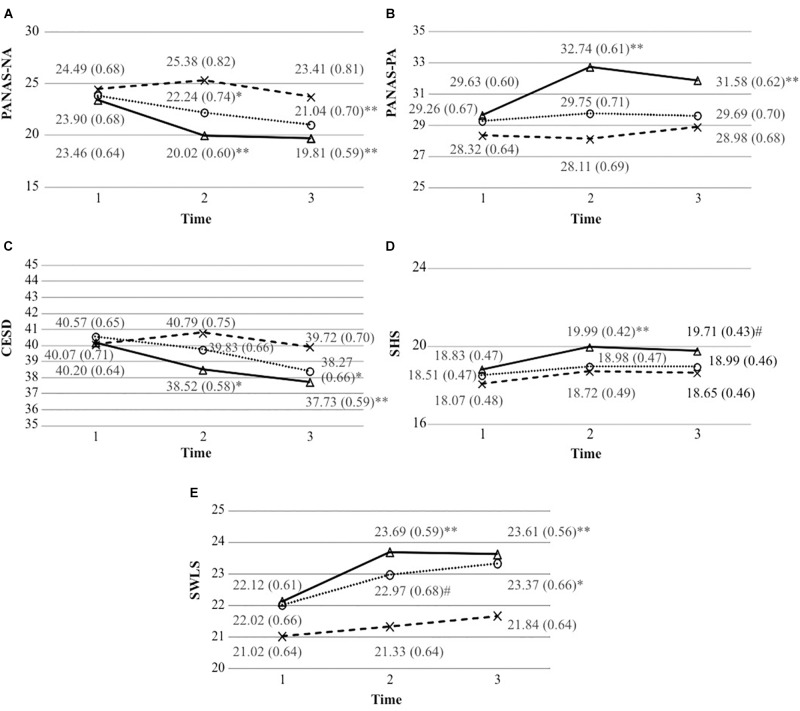
Mean scale scores (and standard errors) of each group at each time period.**(A)** Mean scale scores of the positive and negative affect schedule-negative affect (and standard errors). **(B)** Mean scale scores of the positive and negative affect schedule-positive affect (and standard errors). **(C)** Mean scale scores of the Center for Epidemiological Studies Depression Scale (and standard errors). **(D)** Mean scale scores of the Subjective Happiness Scale (and standard errors). **(E)** Mean scale scores of the Satisfaction With Life Scale (and standard errors). ^#^p < 0.05; ^∗^p < 0.01; and ^∗∗^p < 0.01.

Table 1 shows the characteristics of the sample analyzed. The numerical variables were analyzed through the one factor ANOVA test and the categorical variables through the chi-square test (or Fisher’s exact test). The groups did not differ in any of the variables examined at pretest. Figure 2 presents the means of the scale scores and the standard errors of each group at each time period.

**Table 1:** Characteristics of the sample analyzed at time 1 by allocation group.

Variable (Minimum–Maximum)	Hassles (*n* = 123)	Events (*n* = 134)	Gratitude (*n* = 153)	Total (*n* = 410)	*p*	
			
	Mean (Standard Deviation)^#^				*F* (df)
Age (18–78)	32.64 (10.23)	32.75 (10.47)	32.79 (11.40)	32.73 (10.72)	0.993	0,007 (2, 407)
PANAS negative affect (10–50)^∗^	24.49 (7.62)	23.90 (7.89)	23.46 (7.91)	23.91 (7.81)	0.558	0,585 (2, 407)
PANAS positive affect (10–50)	28.32 (7.15)	29.26 (7.75)	29.63 (7.46)	29.11 (7.47)	0.338	1,088 (2, 407)
CES-D (20–80)	40.07 (7.91)	40.57 (7.51)	40.20 (7.96)	40.28 (7.79)	0.865	0,145 (2, 407)
SHS (4–28)	18.07 (5.35)	18.51 (5.46)	18.83 (5.85)	18.50 (5.57)	0.534	0,628 (2, 407)
SWLS (5–35)	21.02 (7.08)	22.02 (7.67)	22.12 (7.63)	21.76 (7.48)	0.422	0,864 (2, 407)

	**n (%)^##^**				**χ^2^ (df)**

**Sex**					0.676	2,860 (4)
Male	25 (20.3)	30 (22.4)	38 (24.8)	93 (22.7)		
Female	98 (79.7)	104 (77.6)	115 (75.2)	317 (77.3)		
**Region of the Country**					0.859	5,446 (10)
South	57 (46.3)	61 (45.5)	69 (45.1)	187 (45.6)		
Southeast	49 (39.8)	43 (32.1)	56 (36.6)	148 (36.1)		
Other	17 (13.8)	30 (22.4)	28 (18.3)	75 (18.3)		
**Education**					0.914	4,034 (8)
Secondary education	1 (0.8)	2 (1.5)	4 (2.6)	7 (1.7)		
University	56 (45.5)	60 (44.8)	67 (43.8)	183 (44.6)		
Postgraduate	66 (53.7)	72 (53.7)	82 (53.6)	220 (53.7)		
**Marital status**					0.434	3,798 (4)
Single	72 (58.5)	80 (59.7)	87 (56.9)	239 (58.3)		
Married	45 (36.6)	46 (34.3)	50 (32.7)	141 (34.4)		
Separated	6 (4.9)	8 (6.0)	16 (10.5)	30 (7.3)		
**Number of children**					0.273	7,552 (6)
No children	90 (73.2)	108 (80.6)	111 (72.5)	309 (75.4)		
1 child	14 (11.4)	13 (9.7)	24 (15.7)	51 (12.4)		
2 or more children	19 (15.4)	13 (9.7)	18 (11.8)	50 (12.2)		
**Income**					0.737	10,478 (14)
Up to 3 minimum wages^∗∗^	20 (16.3)	28 (20.9)	23 (15.0)	71 (17.3)		
From 3 to 9 minimum wages	57 (46.3)	54 (40.3)	80 (52.3)	191 (46.6)		
From 9 to 15 minimum wages	27 (22.0)	27 (20.1)	25 (16.3)	79 (19.3)		
More than 15 minimum wages	16 (13.0)	19 (14.2)	15 (9.8)	50 (12.2)		
Not stated	3 (2.4)	6 (4.5)	10 (6.5)	19 (4.6)		
**Race/Color**					0.894	2,264 (6)
White	100 (81.3)	105 (78.4)	117 (76.5)	322 (78.5)		
Brown	17 (13.8)	18 (13.4)	24 (15.7)	59 (14.4)		
Other	6 (4.9)	11 (8.2)	12 (7.8)	29 (7.1)		
**Religion**					0.128	12,551 (8)
Catholicism	31 (25.2)	41 (30.6)	47 (30.7)	119 (29.0)		
Spiritism	22 (17.9)	36 (26.9)	24 (15.7)	82 (20.0)		
No religion	40 (32.5)	30 (22.4)	42 (27.5)	112 (27.3)		
Other	30 (24.4)	27 (20.1)	40 (26.1)	97 (23.7)		

*n, sample size; *p*, significance level; df, degrees of freedom; %, percentage; PANAS, positive and negative affect schedule; CES-D, center for epidemiological studies depression scale; SHS, subjective happiness scale; SWLS, satisfaction with life scale. ^#^ANOVA. ^##^Pearson’s Chi-Squared Test. ^∗^Possible minimum and maximum scores in each scale. ^∗∗^The average minimum wage in Brazil at the time of the data collection was BRL 880*.

### Negative Affect

As can be seen in Figure 2A, the Neutral Events and Gratitude groups had lower mean NA scores at time 2 than time 1 (*p* = 0.008 and *p* < 0.001, respectively). A decrease in the mean scores was also observed in both groups in time 3, when compared to time 1 (*p* < 0.001).

At time 2, there were significant differences between the Hassles and the Neutral Events groups (*p* = 0.013, *d* = 0.35) and between the Hassles and the Gratitude groups (*p* < 0.001, *d* = 0.65). At time 3, there were significant differences between the Hassles and the Gratitude groups (*p* = 0.001, *d* = 0.44).

### Positive Affect

According to Figure 2B, in the PA assessment, the Gratitude group experienced an increase in its mean scores on time 2 and time 3, compared to time 1 (*p* < 0.001). At post-test, there were significant differences between the Gratitude and Hassles groups (*p* < 0.001, *d* = 0.60) and between the Gratitude and Neutral Events groups (*p* = 0.004, *d* = 0.38). At time 3, there were significant differences between the Gratitude and the Hassles groups (*p* = 0.014, *d* = 0.34).

### Depression

Figure 2C illustrates that, in the assessment of depression symptoms, the Gratitude group experienced a decrease in mean scores at time 2, when compared with time 1 (*p* = 0.007). At time 3, the decrease in mean scores was maintained in the Gratitude group (*p* < 0.001) and the Neutral Events group also experienced a decrease, in relation to time 1 (*p* = 0.001). The only difference observed between groups was at time 2, between the Gratitude and the Hassles groups (*p* = 0.049, *d* = 0.29).

### Subjective Happiness

Only the Gratitude group had higher mean subjective happiness scores at times 2 and 3, compared to time 1 (*p* < 0.001 and *p* = 0.04, respectively), as shown in Figure 2D. The measurements of the three groups did not differ significantly at time 2 or time 3.

### Life Satisfaction

Examining Figure 2E shows that the Neutral Events and Gratitude groups had higher mean scores on the SWLS scale at time 2 (*p* = 0.011 and *p* < 0.001, respectively) and at time 3 (*p* = 0.001 and *p* < 0.001, respectively) than at time 1. There were significant differences only between the Gratitude and Hassles groups (*p* = 0.019, *d* = 0.33) at time 2.

## Discussion

The main findings of the present study demonstrated that the gratitude intervention was able to increase positive affect, subjective happiness and life satisfaction, and reduce NA and depression symptoms. This change was greater in the Gratitude group than in the control groups for positive affect. In the other dimensions analyzed, similar changes were observed in the gratitude intervention and the neutral events intervention. The most significant changes observed in the Gratitude group were related to the emotional dimension of subjective well-being.

### Intergroup Comparison

With the exception of the results for PA at time 2, the results of the Gratitude group did not have any statistically significant difference in the results in relation to the other scales, when compared to the Neutral Events group. Through the qualitative analysis of the daily lists and spontaneous emails sent by the participants, it was possible to qualitatively interpret these results. For example, the participants from the Neutral Events groups recorded the positive events from their days on the daily lists. This activity was very close to the proposal for the Gratitude group, which may explain the similarity between the results of the two groups. On the other hand, the participants randomized into the Hassles group reported via email that it was disagreeable to recall the problems they had experienced during the day, as though the fact of writing up the list made them remember unpleasant events and relive the same negative emotions felt at the time.

The results of the present study coincide with the findings presented by Dickens (2017) in her series of meta analyses that assessed the effects of gratitude interventions, including gratitude lists, in various dependent variables. To perform the meta analyses, the control groups were divided by the author into three types: neutral interventions (e.g., events list), negative interventions (e.g., hassles list) and positive interventions (e.g., acts of kindness list).

### Comparison With Neutral Interventions

According to the analyses by Dickens (2017), on the post-test, the gratitude intervention group had higher well-being, happiness, PA, and life satisfaction scores and lower scores for depression than the neutral intervention group. There was no difference between the groups in relation to negative affect. In the follow-up, the differences were maintained for well-being, happiness and depression, a small difference was noted for positive affect, and no difference for NA and life satisfaction. In turn, in the present study, at post-test, the scores of the Gratitude group were higher than those of the Neutral Events group for PA only. In the other dimensions – negative affect, subjective happiness, life satisfaction and depression – no difference was observed. In the follow-up, no differences between the two groups were observed in any dimension.

Like the findings of Dickens (2017), in the present study, there was no significant difference in the NA scores in the post-test, when the gratitude intervention group was compared with the neutral intervention group. It is worth mentioning that affect is usually a dimension examined in gratitude intervention studies, but the results are confusing and do not indicate a common trend (Borgueta, 2010). Even in studies where there was a significant difference between the Gratitude group and the comparison group, this difference had a low effect size. It can be assumed that the initial level of affect, or the quality of the instruments used for measuring it, can have an influence (Borgueta, 2010).

### Comparison With Negative Interventions

In the post-test, as also found by Dickens (2017), in the comparison with negative interventions, the gratitude intervention group achieved higher scores for PA and life satisfaction and lower scores for depression and negative affect. In the follow-up, there was no difference for negative affect. In the present study, the Gratitude group had higher scores than the Hassles group for PA and life satisfaction, lower scores for NA and depression and no differences in relation to happiness. At follow-up, the differences for affect, both positive and negative, were maintained.

Dickens (2017) also assessed possible moderating variables in the comparison between gratitude interventions and negative interventions. In relation to age and life satisfaction, it was found that the higher the age, the higher the effect size. Sin and Lyubomirsky (2009) had already pointed out that the benefits from carrying out these activities increased according to age. Dickens supposed that this occurred because adults are more motivated and disciplined than undergraduate students, who generally participate in studies in exchange for credits. In the case of children, gratitude interventions can be very complicated. In general, sex, type of neutral intervention, and follow-up period do not appear to influence effect size.

### Caution in Comparisons With Negative Interventions

The main findings of the present study indicated that gratitude interventions have an impact on well-being, especially on its emotional component (positive affect). In relation to the other dimensions (negative affect, depression symptoms, subjective happiness, and life satisfaction), writing up a daily gratitude list had an impact similar to reporting events that occurred during the day. The measurement of this impact can be observed through effect size. The effect size of the gratitude intervention, when compared with the neutral events list, was average for PA, and small for negative affect, subjective happiness, life satisfaction, and depression symptoms. The gratitude intervention had relatively greater effect sizes when compared with the hassles list: average effect sizes for NA and PA and small effect sizes for depression symptoms, life satisfaction, and subjective happiness.

Various authors have pointed out that there is a risk in comparing the effectiveness of a gratitude intervention with an intervention that is negative in character, such as the Hassles group (Wood et al., 2010; Davis et al., 2016). The difference between a hassles group and a gratitude group may be magnified by the positive effects of a state of gratitude or the negative effects from thinking about stressful and difficult events. In such comparisons, it is not possible to affirm that the improvement observed in a group that performed the gratitude intervention is the result of the intervention *per se* or if it is due to the affliction caused by the negative intervention.

It is very important to take into account with which group the Gratitude group is being compared to be able to interpret the effect size calculated (Dickens, 2017). Generally, larger effect sizes are found when the comparison is made with interventions that analyze negative aspects. For this reason, the inclusion of the Neutral Events group enabled a more cautious interpretation of the data. According to the results, effect size was larger in all dimensions when the Gratitude group was compared with the Hassles group than in the comparison with the Neutral Events group.

One of the main findings of Dickens (2017) was that the effect size of the gratitude intervention group, for most of the variables, was larger when compared with the negative intervention and smaller when compared with the neutral intervention, and inexistent when compared with the positive intervention. This observation is consistent with the warning issued by Wood et al. (2010) that the group with which the gratitude intervention group is being compared influences the interpretation of the results. Most of the effect sizes noted by Dickens (2017) were small or medium; large effect sizes were observed when the gratitude intervention was compared with the negative intervention.

### Limitations of the Study

A limitation inherent in non-pharmacological clinical trials is the impossibility of blinding the participants and appliers of the intervention. For this reason, it is essential to ensure blinding of the outcomes assessment. Another limitation with clinical trials is loss to follow-up. A high dropout rate is expected in self-performed online interventions, due to the absence of human contact and the need for participants to be disciplined, and motivated to carry them out (Wood et al., 2010). Therefore, every effort was made to reduce participant dropouts, such as by sending daily reminders to write the lists. Finally, the analysis demonstrated that there was no difference among losses in the three groups, decreasing the probability of bias affecting the results.

It is worth pointing out that randomized clinical trials in the field of psychology need to be interpreted from a distinct perspective. First of all, the measurements used are complex, since it is not possible to directly measure dimensions that are not easily quantifiable (Espírito-Santo and Daniel, 2015). Second, the results of an intervention may be manifested or start to be manifested in the beginning of the study, but are not present at the end of it, or may take longer and occur after the post-test measurement, since individuals may assign new meaning to the intervention based on new experiences in their lives (Peuker et al., 2009). Finally, the effect sizes of an intervention that is psychological in nature are normally smaller, precisely due to the difficulty of measuring such a subjective difference. As Cohen (1988) pointed out, the classifications established for *d* are relative and must be interpreted according to each context. Therefore, although the effect sizes found in the present study may be considered small, they are of significant clinical relevance, given the nature of the dimensions investigated.

Ensuring the loyalty of participants in an intervention is also difficult (Davis et al., 2016). In an effort to minimize this problem, the participants were requested to send their lists daily, making it possible to monitor who was doing them. This methodology is suggested for future studies. It was explained to the participants that their responses would not be judged or evaluated in terms of being right or wrong, but that monitoring would take place to know whether the intervention was being carried out according to the instructions.

### Future Outlooks

A gratitude list is a simple, cheap, easy, and pleasant intervention to perform. For this reason, it is not contraindicated for any audience, except for rare exceptions, such as participants who may feel bad when noting a feeling of indebtedness toward another person or when recalling a sad event in their lives or thinking about a deceased loved one or someone with whom they have no further contact. Thus, it has the potential to be generalized for various audiences with age range, social and cultural differences, considering the sample of the present study. However, more information is still needed to make gratitude interventions more appropriate for specific groups, such as the best intensity, duration, and format of such interventions, in order to reduce dropout rates and increase the benefits of those who carry them out.

Along the lines of the issue raised by Mitchell et al. (2010), it is also necessary to investigate which characteristics of an intervention ensure better results when the intervention is performed online. Davis et al. (2016) suggest longer and more intense interventions, as well as apply them in clinical samples. Online interventions can primarily target young people, who are more skilled in the use of digital media. However, longer interventions may increase loss to follow-up.

## Data Availability

The datasets generated for this study are available on request to the corresponding author.

## Author Contributions

All authors were involved in the design of the study. LC was responsible for data collection, data analysis, and interpretation of data, supervised by LP and CR. LC wrote the first draft of the manuscript. LP and CR edited the text and contributed to the writing. All authors have approved the final version of the manuscript.

## Conflict of Interest Statement

The authors declare that the research was conducted in the absence of any commercial or financial relationships that could be construed as a potential conflict of interest.
